# Interactive effects of atmospheric oxidising pollutants and heat waves on the risk of residential mortality

**DOI:** 10.1080/16549716.2024.2313340

**Published:** 2024-02-21

**Authors:** Nan Ren, Huimin Huang, Baoying Liu, Chuancheng Wu, Jianjun Xiang, Quan Zhou, Shuling Kang, Xiaoyang Zhang, Yu Jiang

**Affiliations:** a Department of Preventive Medicine, Fujian Provincial Key Laboratory of Environment and Health, School of Public Health, Fujian Medical University, Fuzhou, China; b Department of Public Health, Fuzhou Center for Disease Control and Prevention, Fuzhou, China

**Keywords:** Oxidising pollutants, heat waves, mortality, interactive effects, time-series analysis

## Abstract

**Background:**

The impact of heat waves and atmospheric oxidising pollutants on residential mortality within the framework of global climate change has become increasingly important.

**Objective:**

In this research, the interactive effects of heat waves and oxidising pollutants on the risk of residential mortality in Fuzhou were examined. Methods We collected environmental, meteorological, and residential mortality data in Fuzhou from 1 January 2016, to 31 December 2021. We then applied a generalised additive model, distributed lagged nonlinear model, and bivariate three-dimensional model to investigate the effects and interactions of various atmospheric oxidising pollutants and heat waves on the risk of residential mortality.

**Results:**

Atmospheric oxidising pollutants increased the risk of residential mortality at lower concentrations, and O3 and Ox were positively associated with a maximum risk of 2.19% (95% CI: 0.74–3.66) and 1.29% (95% CI: 0.51–2.08). The risk of residential mortality increased with increasing temperature, with a strong and long-lasting effect and a maximum cumulative lagged effect of 1.11% (95% CI: 1.01, 1.23). Furthermore, an interaction between atmospheric oxidising pollutants and heat waves may have occurred: the larger effects in the longest cumulative lag time on residential mortality per 10 µg/m3 increase in O3, NO2 and Ox during heat waves compared to non-heat waves were [−3.81% (95% CI: −14.82, 8.63)]; [−0.45% (95% CI: −2.67, 1.81)]; [67.90% (95% CI: 11.55, 152.71)]; 16.37% (95% CI: 2.43, 32.20)]; [−3.00% (95% CI: −20.80, 18.79)]; [−0.30% (95% CI: −3.53, 3.04)]. The risk on heat wave days was significantly higher than that on non-heat wave days and higher than the separate effects of oxidising pollutants and heat waves.

**Conclusions:**

Overall, we found some evidence suggesting that heat waves increase the impact of oxidising atmospheric pollutants on residential mortality to some extent.

## Introduction

Climate change and ambient air pollution are regarded as significant global health threats in the 21st century, as they cause significant excess mortality [1,2]. Meanwhile, heat waves and air oxidising pollution [3,4] have been linked to non-accidental deaths in numerous epidemiological studies with time-series analyses [5–7]. Li found that exist positive and significant excess death risks due to the synergism between high temperature and air pollution (PM_10_, O_3_, NO_2_, and CO) in Northeast Asia [6]. Studies also observed an interaction between high temperature and air pollutants (PM_10_, O_3_, NO_2_, and SO_2_) on mortality in China [5,7]. Heat waves and oxidising pollution have independent effects on health [8,9], but their potential interaction could amplify the burden of mortality. A synergistic effect may exist for these two hazards [7]. However, research relating to the potential interactive effects between heat waves and oxidising pollution on mortality is limited today.

Health concerns can arise from both air pollution and extreme heat, and the risk of disease is influenced by the interplay between temperature and oxidising pollutants [10]. Oxidising pollution has been linked to non-accidental deaths in numerous epidemiological studies and been observed in different countries [11–14]. As heat wave events [15,16] have become more frequent in recent years, studies have demonstrated that excessive heat is connected with an increased risk of mortality in the population [17–20]. In developing nations, the number of such studies is rather low, and the results have not yet been harmonised [21].

According to some studies, the combined impacts of high temperatures and air pollution should be more detrimental to health outcomes than each factor acting alone [5–7]. Although the relationship between air pollution and high temperatures has recently received increased attention [22,23], the results are still inconclusive. According to a study conducted in the USA [24], the systemic reaction brought on by short-term ozone exposure varied with temperature and was more severe at high temperatures. Furthermore, other studies in Europe and the USA, where the air quality is good, have also shown an interaction between high temperatures and oxidising pollutants such as photochemical oxidants (such as ozone, O_3_), carbon monoxide (CO) and nitrogen oxides (NO_x_) on the risk of mortality in the population [25–27].

Low levels of oxidising pollutant exposure has been shown to increase the risk of population mortality [28–31], even in Europe, where oxidising pollutant concentrations are better controlled [3,32], while few such studies have been conducted in lightly polluted cities in southern China. Therefore, analysis of the combined effects of heat waves and low-level oxidising pollutant exposure can further inform the development of interventions designed to reduce the burden of oxidising pollutant pollution and heat wave-induced mortality.

Fuzhou is a city characterised by a humid subtropical climate and low level of air pollution in China [33]. Fuzhou is one of China’s hottest provincial capitals according to data from the Chinese Meteorological Agency and has been called an ‘oven’ city because of its extremely hot summers [33]. In comparison to some of the more polluted towns in northern China [34,35], Fuzhou has higher air quality, and many of its air pollutants are below the Chinese National Ambient Air Quality Standard (CAAQS) standard I [36]; this city is considered a low-concentration control site for the National Air Pollution Health Impacts Monitoring Project. Given that China’s air quality is improving as a result of ongoing interventions and that there will be more days with high heat as a result of climate change, Fuzhou was selected as the research location to examine how heat waves and oxidising pollutants in the atmosphere interact. Future research of this kind in other southern coastal cities with hot but less polluted climates may use the findings of this study as a guide.

## Method

### Data collection

We chose Fuzhou City as the study site. The data collected everyday for this study are long time-series of six consecutive years from 1 January 2016, to 31 December 2021, in Fuzhou city; they include environmental data, meteorological data and residential mortality data.

Environmental data were obtained from seven national environmental monitoring sites at the Fuzhou City Environmental Monitoring Centre Station, including daily average concentrations of atmospheric pollutants. Meteorological data were obtained from the Fuzhou Meteorological Bureau, including temperature, barometric pressure and relative humidity. Mortality data were collected by the Fuzhou Centre for Disease Control and Prevention (CDC). Number of daily deaths used the International Classification of Disease version 10 (ICD-10) underlying cause of death codes as the criterion for classifying disease variables and collecting numbered non-accidental total deaths (ICD-10: A00-R99), including deaths due to diseases of the circulatory system, respiratory system, cardiovascular system, cerebrovascular system, etc. The research received ethical approval from Fuzhou Center for Disease Control and Prevention, Bio- and Medical Ethics Committee (No: 2022003). We obtained verbal consent to enter the households and then written informed consent was obtained in the survey to ensure the authenticity of the study.

### Heat wave definition

A heat wave was defined as a consecutive period of at least 3 days during which the daily maximum temperature exceeded the 92.5th percentile of the reference period (2016 − 2021) [37,38]. This definition is optimal for its capacity to capture the real pattern of health effects at a national scale, which is derived from the best model fit of the exposure-response relationship [38,39]. Almost all heat waves occur in warm seasons [9], hence, we limited our research to the period of May to October.

### Data analysis

Studies of the health impacts of temperature and air pollution primarily use time-series studies and case-crossover studies; of these, generalised additive models (GAMs) in time-series research are the most common [40]. A quasi-Poisson connected generalised additive model was used to analyse the effect of exposure factors on population mortality because small probability events, such as deaths roughly follow a Poisson distribution; the variance in daily death counts calculated in this study was higher than the mean.

## Models for the effects of oxidising pollutants on mortality

On the basis of the GAMs, a log-linear exposure-response model was fitted using a spline function [41], and the independent variables were treated non-linearly to plot exposure-response curves, with the following analytical model form:

(1) $$\mathrm{Log}\left(E\left(\mathrm{Yt}\right)\right)=S\left(Z_{t},\mathrm{df}\right)+\mathrm{ns}\left(\mathrm{Time},\mathrm{df}\right)+\mathrm{ns}\left(\mathrm{Xt},\mathrm{df}\right)+\mathrm{Dow}+\mathrm{holiday}+\alpha$$

Where: E (Yt) is the expected value of the number of deaths on day t; Z_t_ is the concentration of atmospheric oxidising pollutant on observation day t; S is a spline function for exposure-response curve fitting; ns()-natural smoothing spline function to control for nonlinear confounding factors; df is its degree of freedom; time is the date variable; Xt is the meteorological factor, including mean temperature, mean barometric pressure and mean relative humidity; Dow is a dummy variable reflecting the day-of-week effect; holiday is a dummy variable reflecting the holiday effect; and ɑ is a constant intercept term.

A single pollutant model was used as the main model to analyse the relative risk of short-term air pollution (O_3_, O_x_, NO_2_) exposure to the number of daily deaths in the population. A multi-day moving average lag01-lag07 was introduced to analyse the cumulative lag effect of pollutants to prevent underestimation of the health effects of pollutants [42,43]. The excess risk and 95% confidence interval (*ER*, *95% CI*) for the risk of mortality when pollutant concentrations were increased were calculated. The specific analytical model form was as follows:

(2) $$\mathrm{Log}\left(E\left(\mathrm{Yt}\right)\right)=\beta Z_{t}+\mathrm{ns}\left(\mathrm{Time},\mathrm{df}\right)+\mathrm{ns}\left(\mathrm{Xt},\mathrm{df}\right)+\mathrm{Dow}+\mathrm{holiday}+\alpha$$

Where: *β* is the exposure response relationship coefficient, i.e. the increase in daily mortality per unit increase in pollutant concentration; other variables are the same as in equation (1).

## Models for the effect of temperature on mortality

The GAMs combined with a distributed lagged non-linear model (DLNM) was used in this study to fit the non-linear temperature-mortality relationship and lagged effects. The maximum lag days were set at 14 days [44]. The analytical model form was as follows:

(3) $$\mathrm{Log}\left(E\left(\mathrm{Yt}\right)\right)=\beta \mathrm{Tem}p_{t,l}+\mathrm{ns}\left(\mathrm{Time},\mathrm{df}\right)+\mathrm{ns}\left(\mathrm{Xt},\mathrm{df}\right)+\mathrm{pollutant}+\mathrm{Dow}+\mathrm{holiday}+\alpha$$

Where: Temp_t,l_ is the lagged days temperature matrix obtained from DLNM; l is the maximum lagged days; pollutant is the explanatory variable that has a linear effect on the dependent variable, which is the concentration of atmospheric pollutants on observation day t; and Xt is relative humidity, which incorporates a low correlation meteorological factor with temperature [45]. Other variables are the same as in equation (1).

## Interaction model for oxidising pollutant and heat waves

A thin-slab spline function was used to demonstrate the interaction between air temperature and atmospheric oxidising pollutants on mortality. Atmospheric oxidising pollutants and temperature were introduced into the model via a continuous equation with bivariate variables, and 3D graphs of these two variables and the ending variables were plotted for visually judging the relationship between the three variables. The model is as follows:

(4) $$\mathrm{Log}\left(E\left(\mathrm{Yt}\right)\right)=\mathrm{TS}\left(P_{\mathrm{tL}},\mathrm{Tem}p_{t}\right)+\mathrm{ns}\left(\mathrm{Time},\mathrm{df}\right)+\mathrm{ns}\left(\mathrm{Xt},\mathrm{df}\right)+\mathrm{Dow}+\mathrm{holiday}+\alpha$$

Where: TS stands for thin-plate regression splines; TS(P_tL_,Tempt) represents the interaction term between atmospheric oxidising pollutants and temperature, P_tL_ is the moving average of atmospheric oxidising pollutants on day t for day L; and Tempt is the daily average temperature on day t. Other variables are the same as in equation (1).

A GAM model was used to assess the risk of oxidising pollutants to mortality under both heat wave and non-heat wave layers. The model is as follows:

(5) $$\mathrm{Log}\left(E\left(\mathrm{Yt}\right)\right)=\beta_{K}Z_{t}H_{K}+\mathrm{ns}\left(\mathrm{Time},\mathrm{df}\right)+\mathrm{ns}\left(\mathrm{Xt},\mathrm{df}\right)+\mathrm{Dow}+\mathrm{holiday}+\alpha$$

Where: *β* is the exposure-response coefficient; H_k_ denotes the dummy variable for heat wave (H_1_ = 0, H_2_ = 0) and non-heat wave days (H_1_ = 0, H_2_ = 0); and *β*_*1*_ and *β*_*2*_ represent the role of oxidising pollutants in the heat wave and non-heat wave layers, respectively. Other variables are the same as in equation (1).

All data analyses were performed using ‘tsModel’, ‘dlnm’, ‘mgcv’, ‘lubridate’, ‘ggplot2’, ‘plyr’ and ‘splines’ package in statistical software R version 4.2.1 [33], *p* = 0.05.

## Results

### Descriptive statistics

Table 1 provides the descriptive statistics on the average number of daily non-accidental deaths and weather conditions in Fuzhou, China, 2016–2021. During the study period, the daily concentrations of PM_2.5_ and PM_10_ were on average 23.06 μg/m^3^ and 44.12 μg/m^3^, respectively, while the daily average values of NO_2_, SO_2_, CO, O_x_ and O_3–8 h_ were 23.36 μg/m^3^, 5.13 μg/m^3^, 0.65 mg/m^3^, 66.36 μg/m^3^ and 88.54 μg/m^3^, respectively. The average barometric pressure, temperature and humidity were 1008.40 hPa, 21.60°C and 72.86%, respectively. The population experiences approximately 32 non-accidental deaths per day.

**Table 1.** Descriptive statistics on daily mortality and weather conditions in Fuzhou, China, 2016–2021.

	Variables	Mean±SD	Min	Max
Daily mortality	Total non-accidental deaths (n)	32 ± 8	12	73
Weather conditions	Daily average temperature (°C)	21.60 ± 6.89	2.60	33.40
	Daily maximum temperature (°C)	26.02 ± 7.55	6.8	40.50
	Daily average relative humidity (%)	72.86 ± 11.89	33.00	100.00
	Daily average barometric pressure (hPa)	1008.40 ± 8.28	983.20	1034.00
Environmental conditions	PM_2.5_ (μg/m^3^)	23.06 ± 12.00	2.50	83.38
	PM_10_ (μg/m^3^)	44.12 ± 20.60	5.25	167.57
	NO_2_ (μg/m^3^)	23.36 ± 10.50	3.57	79.57
	O_3–8 h_ (μg/m^3^)	88.54 ± 32.06	16.71	208.43
	O_x_ (μg/m^3^)	66.36 ± 21.00	20.75	147.38
	SO_2_ (μg/m^3^)	5.13 ± 1.60	2.14	18.57
	CO (mg/m^3^)	0.65 ± 0.14	0.32	1.73

## Effects of oxidising pollutants on mortality

**Figure 1.**
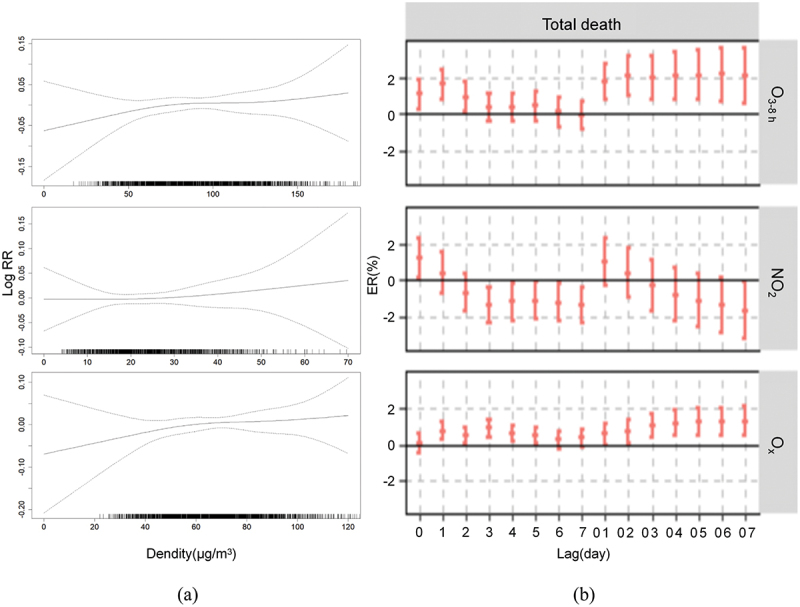
The impact of oxidising pollutants on the health of the population. (a) Exposure-response curves for O_3_, NO_2_ and O_x_ on the main causes of mortality in the population. (b) Impact of each 10 μg/m^3^ increase in O_3_, NO_2_ and O_x_ on the risk of excess mortality in the population (*ER* values and *95% CI*).

Figure 1(a) shows the curves of O_3_, NO_2_ and O_x_ and the risk of mortality in Fuzhou. There was a positive correlation between increasing concentrations of O_3_, NO_2_ and O_x_ and the relative risk of death for the population (*p* < 0.05). The curves for the effects of O_3_ and NO_2_ on mortality are approximately linear and increase at the smallest concentrations, O_3_: 70 ~ 100 µg/m^3^ and NO_2_: 15 ~ 25 µg/m^3^.

O_3_, NO_2_ and O_x_ were introduced as linear independent variables for the single pollutant model analysis. Figure 1(b) and Supplement Table 1 show the effect of each 10 μg/m^3^ increase in O_3_, NO_2_ and O_x_ concentrations on the excess mortality risk of residents in Fuzhou. The results show a more significant positive association overall between O_3_ and resident mortality, for which the overall O_3_ effect value [2.13% (0.6%, 3.68%)] is higher than that of O_x_ [1.29% (0.51%, 2.08%)]; a more significant negative association is found between NO_2_ and resident mortality, with an effect value [−1.67% (−3.23%, −0.09%)]. O_3_, O_x_ and NO_2_ have different effect values on mortality and show inconsistent lagging characteristics.

## Effects of heat waves on mortality

There were 24 heat wave events in Fuzhou during May–October 2016–2021, with a total of 114 heat wave days and 990 non-heat wave days. Heat waves occurred as early as mid-June and as late as late-September. 2020 had the highest number of heat wave events at 6. 2020 and 2021 had the highest number of heat wave days at 27. 2017 and 2018 had the longest duration of heat wave events at 10 days.

**Figure 2.**
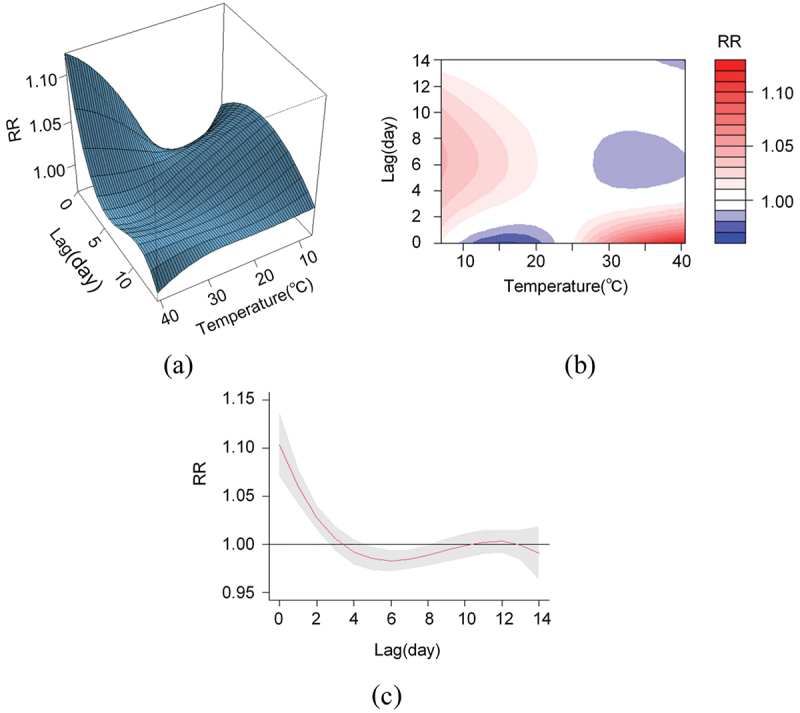
The impact of temperature on the health of the population. (a) 3D maps of the effect of temperature on residential mortality. (b) Contour maps of the effect of temperature on residential mortality. (c) Cross-sectional plot of the impact of heat waves on residential mortality (*RR* values and *95% CI*).

Figure 2(a,b) shows the 3D and contour plots of the effect of temperature on mortality, respectively. We can see a significant positive effect of temperature on residential mortality at high versus low temperatures, while the risk value is greater in extremely hot weather and on an upward trend.

A daily maximum temperature above 92.5% for three consecutive days was used as a cutoff point to classify heat wave days and non-heat wave days. Figure 2(c) and Supplement Table 2 show the cut-plot and relative risk (*RR)* values of heat wave days on residential deaths in Fuzhou City at different lag days from 2016 to 2021. The *RR* of the impact of the heat wave on total non-accidental resident deaths reached a maximum on the day of the heat wave at 1.10% (1.07%, 1.14%); its impact persisted for 2–3 days and then disappeared (*p* < 0.05). And it also presents a higher risk at 1.11% (1.01%, 1.23%) in cumulative effects.

## Effects of oxidising pollutants interacting with heat waves on mortality

**Figure 3.**
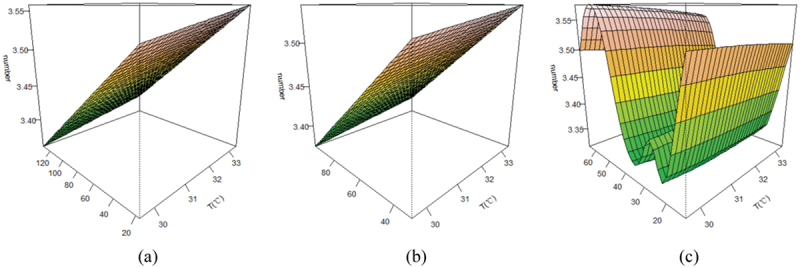
Response to the effects of oxidising pollutants and temperature in major mortality. (a): O_3_, (b): O_x_, (c): NO_2_.

Figure 3 shows a bivariate three-dimensional surface plot of the effects of oxidising pollutants and heat wave day temperature on the main causes of death. We found that the effects of oxidising pollutants on residential mortality were not entirely consistent at heat wave day temperature levels. The number of deaths increased with increasing concentrations of oxidising pollutants O_3_ and O_x_ and temperature; the maximum number of resident deaths was reached when both oxidising pollutant concentrations and temperature were at their maximum; NO_2_ had a strong effect at low pollutant concentrations, and the concentration played a major role.

**Figure 4.**
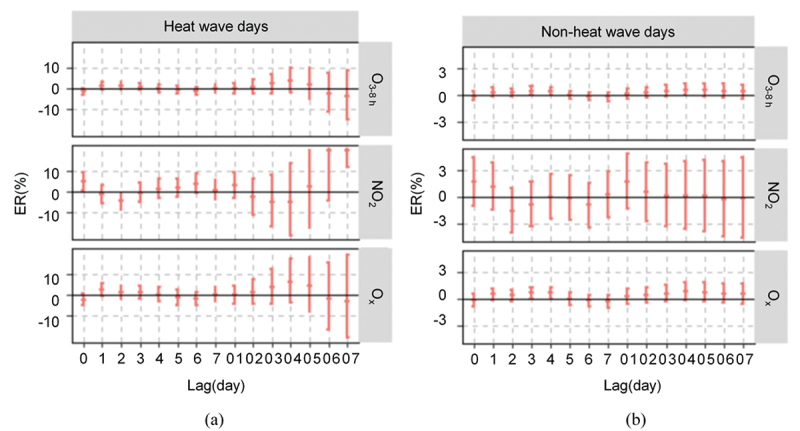
The impact of oxidising pollutants and heat waves on the health of the population. (a) and (b) are excess risk lag distribution of each 10 μg/m^3^ increase in O_3_, O_x_ and NO_2_ on the excess risk of mortality during heat wave and non-heatwave days, respectively (*ER* values and *95% CI*).

Figure 4 and Supplement Table 3 present the current day lagged (lag0) and continuous cumulative (lag0–7) effects of each 10 μg/m^3^ increase in oxidising pollutant concentrations on excess residential mortality effects during the heat wave and non-heat wave periods. Compared to non-heat wave periods, O_3_ and O_x_ had significantly increased effects on cerebrovascular disease deaths on heat wave days and interacted with the heat wave, with lag0–7 risk (*ER*) values of −3.81% (−14.82%, 8.63%) and −3.00% (−20.80%, 18.79%), respectively; these values were significantly higher than those on non-heat wave days, for which no statistically significant difference was found. NO_2_ significantly increased the risk of excess mortality in the population on both heat wave and non-heat wave days; higher risks occurred on heat wave days, with lag0–7 risk (*ER)* values of 67.9% (11.55%, 152.71%) and 16.37% (2.43%, 32.20%), indicating a statistically significant difference. By calculating the upper limit of O_3_,NO_2_,O_x_ attributable risk due to the heatwave can be up to 6.82%, 120.51%, 15.75%.

## Discussion

In our study, the effects of atmospheric oxidising pollutants and heat waves on the risk of residential mortality in Fuzhou were analysed by distributed lag nonlinear models and generalised additive models. Our research found a potential interactive effect of temperature and higher oxidising pollutant concentrations on the increased risk of resident deaths for which temperature played a major role, with the risk increasing with increasing temperature.

The evidence that oxidised pollutants increase the risk of population mortality is already strong. Our study found a positive correlation between increased O_3_, O_x_ and NO_2_ and the relative risk of death in the population, and NO_2_ remained relevant even at low concentrations. Studies in the USA found a positive correlation between O_3_ and the relative risk of total non-accidental mortality, and this correlation persisted at background concentrations [11,46], which is consistent with the findings of this study. In addition, our study suggested that low concentrations of O_3_ and NO_2_ still have a significant effect on mortality, and short-term exposure to oxidising pollutants can increase the risk of mortality. The cumulative lagged effect of air oxidising pollutants on the daily number of residential deaths in this study was stronger than the daily lagged effect, which is consistent with the findings of earlier studies [21,47].

Currently, in addition to research on oxidising pollutants, much interest has been given to the study of non-accidental deaths from heat waves. According to this study, the risk of death increased more noticeably and gradually as a result of high temperatures. High temperatures had an impact on residents’ likelihood of dying on all lag days, with the same day and second lag days having the biggest impact. The favourable effect persisted at a total lag of 14 days, with the largest lag occurring between the first and second day of the lag. This pattern is in line with the findings of the study by Berhanu et al. [48], which found that high temperatures put a greater strain on people’s health.

As the climate crisis progresses, high temperatures and air pollution are expected to increase, with a high probability of occurring simultaneously, and health impacts will synergistically worsen beyond the sum of the individual effects of temperature and air pollution [49]. The bivariate 3D model revealed that the interaction between O_3_ and O_x_ concentrations and temperature during heat waves was not significant for the risk of death, but the risk of death tended to increase with increasing temperature. While a study in Germany on the relationship between daily temperature and cause-specific mortality did not find an interaction between temperature and O_3_ [50], a time-series study in China did find a relationship between hot weather and O_3_ on the risk of death from cerebrovascular disease in the population [6].

In addition, the interaction results showed a synergistic effect between high temperatures and oxidising pollutants, although these results were not statistically significant. This finding is consistent with a previous study [49]. But from the first to the third day of the lag between the heatwave interaction and the oxidative pollutant contaminant, we found that the risk was higher than on the non-heatwave days. On heat wave days, the body’s respiratory system becomes more taxed. As a result of the increased respiratory effort required in hot weather, more pollutants are inhaled, indirectly raising the human body’s exposure level. Because the biological mechanisms behind the effects of heat and pollution on human health are similar, the combined effects of exposure to both elements may be more detrimental to health than the effects of either element alone. For instance, O_3_ and O_x_ can increase platelet activation and blood pressure, suggesting a possible mechanism by which ozone may affect cardiovascular health [51]. During heat wave days, the rise in temperature causes an increase in blood viscosity and cholesterol concentration, which is manifested as an increase in blood pressure [52], which then increases blood flow in the skin; this situation exacerbates suffering from cardiovascular and cerebrovascular diseases [53]. Therefore, both high temperature and oxidising pollutants can create oxidising stress and vascular damage, harming cardiovascular health and increasing the chance of death.

The interaction of heat waves and NO_2_ is also worth discussing. The bivariate 3D model analysis findings revealed that both higher and lower NO_2_ concentrations increased the risk of mortality for local residents, with the effect growing with higher concentrations; as temperature increased, the risk of mortality showed a slight decrease but still had a generally positive impact on mortality risk. The impact of heat wave days was further examined, and NO_2_ was discovered to increase the additional mortality risk on both heat wave and non-heat wave days. This finding is consistent with the results of a survey in China [54] and a meta-analysis [55] that did not detect an effect of temperature on NO_2_-related effects. Nevertheless, Corso et al.‘s findings [56] also suggest that NO_2_ raises only the risk of mortality and excess mortality in hot weather. The study results are varied.

High temperatures, O_3_ and NO_2_ might truly work in concert. High temperatures, prolonged sunshine, and other meteorological factors aid the production of ozone [57], the primary photochemical pollutant, which is created when atomic and molecular oxygen mix quickly. Ultraviolet radiation causes NO_2_ to breakdown into nitrogen oxides (NO) and atomic oxygen, a result of the photochemical production process of NO_2_ [58]. In this situation, ozone can also react with NO to form NO_2_, which is another source of NO_2_ [59]. Additionally, photochemical reactions release secondary organic aerosols that increase inflammatory responses, thus raising the risk of circulatory disease and health damage [60].

In contrast to the results of previous studies of highly polluted areas [34,35], the results of this study reveal the interaction of heat waves and oxidising pollutants in cities with relatively low pollution levels and relatively high temperatures in China. However, there are non-significant results, may not be sufficiently persuasive, and we will continue to observe and improve our study in subsequent studies. This study may have exposure measurement errors because the analysed data, including temperature and oxidising pollutant concentrations, came from outdoor monitors, whereas people usually spend more time indoors in hot weather. A review shows proportional differences in indoor and outdoor concentrations of ozone [61]. The results of the interaction effect may also be influenced by factors such as the socioeconomic level and behavioural habits of the study population because different geographical locations result in different climatic environments [62,63]. Therefore, interactions should be studied from as many angles as feasible.

## Conclusion

In our study, exposure to heat waves and oxidising air pollutants were individually increased mortality risk; in addition, simultaneous exposure to both risk factors had a greater mortality impact than the sum of their individual effects, and that there is a stronger synergy between heat wave days and oxidative pollutants on humans than on non-heat wave days. Given that the frequency of heat waves and high pollution days is expected to increase as climate change progresses, assessing the health impacts of simultaneous exposure to air pollution and extreme heat events is urgent to better guide adaptation policies and interventions that can reduce mortality during extreme heat and oxidising pollution days.

## Supplementary Material

supplements_.docx

## Data Availability

Due to the nature of the research, due to ethical supporting data is not available.

## Paper context

As we known, oxidising pollutants and heat waves could both raise the risk of mortality. Then, we stratified temperature and found that the risk on heat wave days was significantly higher than non-heat wave days. In addition, simultaneous exposure to both risk factors had a greater mortality impact than the sum of their individual effects. The results may suggest interventions in southern coastal cities with hot but less polluted climates.
