# Supplementary material for: Interactive effects of atmospheric oxidising pollutants and heat waves on the risk of residential mortality
Source: Glob Health Action. 2024 Feb 21;17(1):2313340. doi: 10.1080/16549716.2024.2313340 (PMC10883108; doi:10.1080/16549716.2024.2313340)
Supplement: supplements_.docx [file ZGHA_A_2313340_SM0015.docx]

| Supplement Table 1 Impact of oxidizing pollutants on the risk of mortality (*ER* values and *95%* *CI*) | | | |
| --- | --- | --- | --- |
|  | O_3_ | NO_2_ | O_x_ |
| Lag0 | 1.09 (0.24, 1.95)^*^ | 1.22 (0.12, 2.33)^*^ | 0.11 (-0.44, 0.65) |
| Lag1 | 1.65 (0.82, 2.49)^*^ | 0.4 (-0.71, 1.53) | 0.71 (0.22, 1.2)^*^ |
| Lag2 | 0.95 (0.14, 1.77)^*^ | -0.7 (-1.72, 0.33) | 0.5 (0.03, 0.98)^*^ |
| Lag3 | 0.36 (-0.44, 1.18) | -1.34 (-2.34, -0.33)^*^ | 0.88 (0.41, 1.35)^*^ |
| Lag4 | 0.38 (-0.43, 1.19) | -1.19 (-2.18, -0.18)^*^ | 0.59 (0.13, 1.06)^*^ |
| Lag5 | 0.44 (-0.36, 1.24) | -1.12 (-2.11, -0.11)^*^ | 0.50 (0.03, 0.97)^*^ |
| Lag6 | 0.12 (-0.68, 0.93) | -1.2 (-2.2, -0.19)^*^ | 0.24 (-0.23, 0.7) |
| Lag7 | -0.05 (-0.85, 0.75) | -1.38 (-2.37, -0.38)^*^ | 0.34 (-0.12, 0.81) |
| Lag0-1 | 1.8 (0.83, 2.79)^*^ | 1.05 (-0.22, 2.34) | 0.58 (-0.02, 1.17) |
| Lag0-2 | 2.07 (0.98, 3.17)^*^ | 0.41 (-0.94, 1.78) | 0.72 (0.09, 1.35)^*^ |
| Lag0-3 | 2.05 (0.86, 3.25)^*^ | -0.32 (-1.72, 1.1) | 1.03 (0.37, 1.69)^*^ |
| Lag0-4 | 2.08 (0.8, 3.38)^*^ | -0.77 (-2.22, 0.69) | 1.16 (0.47, 1.86)^*^ |
| Lag0-5 | 2.17 (0.81, 3.56)^*^ | -1.11 (-2.6, 0.4) | 1.25 (0.53, 1.98)^*^ |
| Lag0-6 | 2.19 (0.74, 3.66)^*^ | -1.37 (-2.9, 0.18) | 1.25 (0.50, 2.01)^*^ |
| Lag0-7 | 2.13 (0.6, 3.68)^*^ | -1.67 (-3.23, -0.09)^*^ | 1.29 (0.51, 2.08)^*^ |
| Excess risk lag distribution of each 10μg/m^3^ increase in O_3_, O_x_ and NO_2_ on the risk of mortality.  ^*^ : It indicates statistically significant results based on the 95% empirical confidence interval, *p* < 0.05. | | | |

| Supplement Table 2 Impact of heat waves on the risk of mortality (*RR* values and *95%* *CI*) | | | |
| --- | --- | --- | --- |
|  | *RR* |  | *RR* |
| lag0 | 1.10 (1.07, 1.14)^*^ | Lag8 | 0.99 (0.98, 1.00) |
| lag1 | 1.06 (1.04, 1.08)^*^ | Lag9 | 0.99 (0.98, 1.01) |
| lag2 | 1.03 (1.02, 1.04)^*^ | Lag10 | 1.00 (0.99, 1.01) |
| lag3 | 1.01 (0.99, 1.02) | Lag11 | 1.00 (0.99, 1.01) |
| lag4 | 0.99 (0.98, 1.00) | Lag12 | 1.00 (0.99, 1.01) |
| lag5 | 0.98 (0.97, 1.00) | Lag13 | 1.00 (0.98, 1.02) |
| lag6 | 0.98 (0.97, 0.99)^*^ | Lag14 | 0.99 (0.96, 1.02) |
| lag7 | 0.98 (0.97, 0.99)^*^ | lag0-14 | 1.11 (1.01, 1.23)^*^ |
| ^*^ : It indicates statistically significant results based on the 95% empirical confidence interval, *P* < 0.05. | | | |

| Supplement Table 3 Impact of oxidizing pollutants and heat waves on the risk of mortality (*ER* values and *95%* *CI*) | | | | | | |
| --- | --- | --- | --- | --- | --- | --- |
|  | O_3_ | | NO_2_ | | O_x_ | |
|  | Heat waves | Non-heat waves | Heat waves | Non-heat waves | Heat waves | Non-heat waves |
| lag0 | -1.6 (-3.3, 0.13) | 0.83 (-0.66, 2.34) | 4.76 (0.27, 9.45)^*^ | 2.69 (-4.88, 10.86) | -2.36 (-5.24, 0.62) | 1.23 (-0.93, 3.44) |
| lag1 | 1.19 (-0.55, 2.97) | 0.23 (-1.15, 1.63) | -1.31 (-5.6, 3.17) | 3.91 (-3.53, 11.94) | 2.17 (-0.87, 5.30) | 0.43 (-1.59, 2.49) |
| lag2 | 0.98 (-0.78, 2.78) | 0.71 (-0.62, 2.06) | -4.66 (-8.67, -0.47)^*^ | -1.44 (-8.32, 5.96) | 1.18 (-1.90, 4.35) | 0.98 (-0.98, 2.99) |
| lag3 | 0.68 (-1.07, 2.46) | -0.32 (-1.64, 1.01) | -0.49 (-4.81, 4.02) | -0.33 (-7.21, 7.06) | 1.26 (-1.76, 4.37) | -0.47 (-2.40, 1.51) |
| lag4 | -0.04 (-1.88, 1.82) | -0.61 (-1.92, 0.72) | 1.25 (-3.09, 5.78) | 4.59 (-2.64, 12.36) | 0.14 (-3.07, 3.45) | -0.76 (-2.70, 1.21) |
| lag5 | -0.85 (-2.81, 1.14) | -0.61 (-1.92, 0.72) | 1.73 (-2.54, 6.19) | 6.44 (-0.95, 14.39) | -1.39 (-4.83, 2.17) | -0.72 (-2.65, 1.25) |
| lag6 | -1.38 (-3.17, 0.44) | -0.58 (-1.89, 0.75) | 3.7 (-0.78, 8.4) | 13.47 (5.65, 21.88)^*^ | -2.16 (-5.27, 1.05) | -0.52 (-2.45, 1.45) |
| lag7 | -0.09 (-2.00, 1.86) | -1.02 (-2.33, 0.31) | 0.61 (-3.89, 5.33) | 12.04 (4.29, 20.37)^*^ | -0.07 (-3.41, 3.39) | -1.18 (-3.11,0.79) |
| Lag0-1 | -0.37 (-2.79, 2.11) | 0.67 (-0.98, 2.35) | 2.76 (-3.4, 9.31) | 4.53 (-4.17, 14.02) | -0.3 (-4.49, 4.08) | 1.05 (-1.34, 3.50) |
| Lag0-2 | 0.74 (-2.63, 4.22) | 0.94 (-0.82, 2.74) | -2.77 (-11.06, 6.29) | 2.54 (-6.73, 12.74) | 1.05 (-4.77, 7.22) | 1.38 (-1.18, 4.02) |
| Lag0-3 | 2.17 (-2.39, 6.95) | 0.64 (-1.23, 2.54) | -5.21 (-16.72, 7.90) | 1.97 (-7.85, 12.84) | 3.59 (-4.25, 12.07) | 0.94 (-1.77, 3.73) |
| Lag0-4 | 3.56 (-2.34, 9.81) | 0.28 (-1.67, 2.27) | -5.21 (-21.00, 13.74) | 4.03 (-6.59, 15.85) | 6.28 (-3.78, 17.4) | 0.49 (-2.35, 3.41) |
| Lag0-5 | 1.95 (-5.19, 9.62) | 0.03 (-2.01, 2.11) | 2.03 (-17.38, 25.99) | 6.42 (-5.07, 19.3) | 4.00 (-8.08, 17.66) | 0.19 (-2.78, 3.25) |
| Lag0-6 | -2.48 (-11.55, 7.54) | -0.13 (-2.26, 2.04) | 26.58 (-4.29, 67.40) | 11.54 (-1.16, 25.88) | -1.95 (-16.84, 15.60) | 0.06 (-3.04, 3.27) |
| Lag0-7 | -3.81 (-14.82, 8.63) | -0.45 (-2.67, 1.81) | 67.9 (11.55, 152.71)^*^ | 16.37 (2.43, 32.20)^*^ | -3.00 (-20.80, 18.79) | -0.3 (-3.53, 3.04) |
| Under the different temperature levels, excess risk lag distribution of each 10 μg/m^3^ increase in O_3_, O_x_ and NO_2_ on the excess risk of mortality.  ^*^ : It indicates statistically significant results based on the 95% empirical confidence interval, *P* < 0.05. | | | | | | |
